# Copenhagen infant mental health project: study protocol for a randomized controlled trial comparing circle of security –parenting and care as usual as interventions targeting infant mental health risks

**DOI:** 10.1186/s40359-016-0166-8

**Published:** 2016-11-22

**Authors:** Mette Skovgaard Væver, Johanne Smith-Nielsen, Theis Lange

**Affiliations:** 1 Department of Psychology, University of Copenhagen, Øster Farimagsgade 2A, 1353 Copenhagen K, Denmark; 2 Section of Biostatistics, University of Copenhagen, Øster Farimagsgade 5, 1014 Copenhagen K, Denmark

**Keywords:** Indicated intervention, Parenting education, Early intervention, Preventive intervention, Maternal sensitivity, Postnatal depression, Infant social withdrawal, Attachment, Community health services

## Abstract

**Background:**

Infant mental health is a significant public health issue as early adversity and exposure to early childhood stress are significant risk factors that may have detrimental long-term developmental consequences for the affected children. Negative outcomes are seen on a range of areas such as physical and mental health, educational and labor market success, social network and establishing of family. Secure attachment is associated with optimal outcomes in all developmental domains in childhood, and both insecure and disorganized attachment are associated with a range of later problems and psychopathologies. In disadvantaged populations insecure and disorganized attachment are common, which points to the need of identifying early risk and effective methods of addressing such problems. This protocol describes an experimental evaluation of an indicated group-based parental educational program, Circle of Security–Parenting (COS-P), currently being conducted in Denmark.

**Methods/design:**

In a parallel randomized controlled trial of two intervention groups this study tests the efficacy of COS-P compared to Care as Usual (CAU) in enhancing maternal sensitivity and child attachment in a community sample in the City of Copenhagen, Denmark. During the project a general population of an estimated 17.600 families with an infant aged 2–12 months are screened for two known infant mental health risks, maternal postnatal depression and infant social withdrawal. Eligible families (*N* = 314), who agree to participate, will be randomly allocated with a ratio of 2:1 into the COS-P intervention arm and into CAU. Data will be obtained at inclusion (baseline) and at follow-up when the child is 12–16 months. The primary outcome is maternal sensitivity. Secondary outcomes include quality of infant attachment, language, cognitive and socioemotional development, family functioning, parental stress, parental mentalizing and maternal mental wellbeing.

**Discussion:**

The potential implications of the experimental evaluation of an indicated brief group-based parenting educational program to enhance parental sensitivity and attachment are discussed.

**Trial registration:**

ClinicalTrials.govID: NCT02497677. Registered July 15 2015

## Background

Infant mental health is a significant public health issue. Extensive research has shown that early adversity and exposure to early childhood stress are significant risk factors that may have detrimental long-term developmental consequences for the affected children. Negative outcomes are seen on a range of areas such as physical and mental health, educational and labor market success, social network and establishing of family [1, 2]. Young infants are more socially invisible than older children and they are completely dependent on their caregivers for their survival, which make them more vulnerable and exposed to mental health risks [3]. Infants may be at risk due to a particular biological risk (e.g. infantile autism, retardation, prematurity, physical disabilities etc.) or to psycho-social risks in the family (e.g. mentally ill parents, poverty, drug/alcohol abuse etc.). In Denmark the most recent estimates indicate that one in five families is at risk of inadequate parenting abilities/resources and child neglect [4] and 0.05 per thousand children are at risk of terminal child maltreatment [5].

There is by now solid evidence that the establishment of attachment relationships, i.e. a stable emotional bond with a caregiver-mostly the parent-is one of the most important developmental milestones in infancy. Early parent–child attachment relationships function as a blueprint for future social relationships and serve as a framework within which children learn how to deal with stressful situations and to regulate the accompanying negative emotions [6]. Insecure and disorganized attachment is a significant risk for longitudinal child development and psychopathology, as the ability to regulate ones feelings of stress and negative emotions is important for a wide range of socio-emotional outcomes ranging from social competence [7], moral development and empathy [8] to academic achievement [9]. Recent meta-analyses show that insecure and disorganized children have a higher risk of developing mental problems later in life. Insecurely attached children are more likely than securely attached children to develop internalizing problems, such as anxiety and depressive symptoms [10], as well as externalizing problems such as aggressive behavior [11]. For externalizing problems, the risk is even higher for disorganized children [11]. Furthermore, research indicate that severe stress caused by neglect and inadequate parenting during a child’s early years may become “toxic” and impact physiological processes to disturb early brain development [12, 13].

Evidence from attachment research shows that sensitive parenting, where the parent is alert and able to understand the infant’s expression of emotional states and able to manage and meet the infant’s needs contingently, adequately and in a comforting way will lead to the establishment of a pattern of secure attachment in the child. Lack of availability, inconsistent availability, misunderstanding of the infant’s emotional expression and parental behavior that frightens the infant may all lead to an insure attachment (avoidant or ambivalent/resistant) and in the most severe cases a disorganized attachment. This is indicative of a breakdown of an organized (secure or insecure) attachment behavioral strategy. Disorganized attachment is considered to be the result of parental behavior that is frightening for the child [14]. An extreme example of such behavior is child maltreatment, but all sorts of parental behavior that are not comprehensible for the child, such as dissociation, which is common in depressed parents, is potentially frightening for the child. Parental behavior that is frightening for the child results in an emotional dilemma and the paradoxical situation that the parent at the same time is a source of comfort and a source of fear. Thus, in stress situations the child does not know what to do or whom to turn to for comfort and protection, and the behavioral strategy collapses.

Attachment research over the last thirty years has shown that in typical populations the prevalence of securely attached children is only around two thirds, avoidant insecure attachment is seen in one out of five children and insecure ambivalent/resistant is seen in one out of seven children [15, 16]. The prevalence of disorganization ranges from 13 to 82% depending on the presence and type of family risk factors [17]. In disadvantaged populations insecure and disorganized attachment has a prevalence up to 40%, and in the group of neglected and in particular maltreated children the prevalence of disorganized attachment may be as high as up to 80% [18].

Within attachment research the quality of the parent–child attachment relation is typically measured using the gold standard method developed by Mary Ainsworth, the Strange Situation Procedure (SSP), when the child is 12–24 months old [19, 20]. However, research indicates that risk of attachment disturbances may be possible to detect already during the first year of the child’s life. Infant social withdrawal indicates infant distress and it is suggested that this may be indicative of early attachment disturbances and it has been found to be a serious risk factor for infant mental health [21, 22]. Infant social withdrawal is seen by lack of either positive emotional expressions (e.g. smiling, vocalizing, eye contact) or negative protestations (e.g. crying, fussiness, frowning). According to Dollberg, Feldman, Keren, and Guedeney [23] sustained withdrawal behavior in infants can be seen as a more chronic diminishing of the attachment system, which over time may develop in to a generalized persistent pattern of lowered engagement and reactivity to the environment. In more European countries the use of the validated systematic screening method, Alarm Distress Baby Scale (ADBB) [24] for identifying infant delayed socio-emotional development in infant mental health clinics and in home visiting programs have shown promising results [22, 25, 26].

The ADBB is an observational instrument with 8 items related to the infant’s social behavior. It is used during a routine physical examination of the infant aged 2–24 months where the clinician, e.g. the healthcare nurse, engages with the infant. The 8 items, each rated from zero to four are: facial expression; eye contact; general level of activity; self-stimulation gestures; vocalizations; briskness of response to stimulation; relationship to the observer, and attractiveness to the observer. The clinician keeps the 8 items in mind while conducting the routine physical assessment, and then spends approximately 5 min completing the scale. Low scores indicates optimal social behavior, and a cut-off score of five is recommended. In infant cases scoring five or more, the ABDD observation is recommended to be conducted again after 2 weeks to assess whether the social withdrawal is persistent [24]. The prevalence of socially withdrawn infants has in more studies been found to be 3–4% [25, 27]. This points to the possibility of using the ADBB for identification of infant mental health risk and for early intervention to promote the development of a secure attachment relation. The ADBB is described in more details in the Method section.

Postnatal depression (PND) is a another well-known risk for infant mental health. A meta-analysis shows that up to 19% of new mothers may experience minor or major depression during the first months after giving birth. If only including major depression, the prevalence was found to be 7.1% [28]. In a more recent European study, 1,066 women were followed from pregnancy to 12 months postpartum [29]. The results indicated that 9.6% of new mothers may experience a major depressive episode during the first year after delivery. No estimates were given for minor depression. Most cases develop within the first 3 months with a peak incidence of about 4–6 weeks [30, 31].

PND has been found to negatively impact on the mother, her partner [32] her family [33], mother-baby interactions and quality of the attachment relation [34–37] and the longer term socio-emotional and cognitive development of the child [38–40]. It has repeatedly been shown that compared to non-depressed mothers, depressed mothers are more irritable and hostile, less engaged, exhibit less emotion and warmth and they are less sensitively attuned to their infants, which implicates that depressed mothers are less able to appropriately respond to their children’s needs [19, 41]. Those early disruptions in mother-infant interaction may have long-term negative consequences for children’s development. Infants of depressed mothers compared to infants of non-depressed mothers have been found to show more negative behaviors such as social withdrawal, more gaze and head aversion, less expression of positive affect and more expression of negative affect when interacting with their mothers [22, 42, 43]. Such negative ongoing mother-infant behaviors may initiate persistent negative and maladaptive interaction cycles, where the infant is withdrawing from contact and emotional communication, which again contributes to an increase of the mother’s experience of stress [44]. Extensive evidence from attachment research shows that low maternal sensitivity and maladaptive mother-infant interactions are significant risk factors for the child developing an insecure or disorganized attachment pattern [44].

The Edinburg Postnatal Depression Scale, EPDS [45] is a well validated self-report questionnaire for detection of women at risk for or suffering from PND at a clinical level. Across countries the EPDS has been shown to have a high sensitivity (68–95%) and high specificity (78–96%) against a clinical psychiatric diagnosis of depression [46–50]. EPDS comprises questions with 4 possible responses related to mood and feelings. Total score ranges from 0 to 30 points. Scores in the range of 0–9 are considered as indicating the presence of symptoms of distress that may be short-lived. Scores from 10 to 12 are considered to indicate probable depression, and further assessment is recommended. Scores equal to or above 13 are considered to indicate the presence of depression [45, 50]. For more details of the EPDS see measures.

Mothers with PND are often treated individually for their depression. For example, they may receive medical treatment or individual psychotherapy. However, even when the depression is effectively treated, this effect does not necessarily transfer into an improvement of the quality of the mother-infant relation or the cognitive and socioemotional development of the child [51–53]. This points to the need for interventions that focus on supporting mothers with postnatal depression in promoting sensitive interacting and relating to their infants [54, 55]. Further, it has been found that treating mothers with postnatal depression in groups is effective, as the participants face some of the same challenges. The group setting contributes to reduce isolation and stigma for the women, as it provides a network and a mutual learning environment as well as it enables a number of women to be treated at once [56, 57].

### Rational of the circle of security – parenting intervention

Recently, building on evidence from attachment research, a special focus is given to preventive group programs that enhance parental sensitivity and secure attachment. A recent review study concludes that a number of interventions appear to be effective in improving attachment [18]. One of these programs is the intervention program “Circle of Security (COS)” [58]. Based on findings from more studies, COS has proved efficient in enhancing secure attachment as well as reducing maternal depressive symptomatology in high-risk samples, including mothers in prison and mentally ill mothers [58–61]. The original COS program consists of 20 weekly sessions of 2 h durations and includes an initial video assessment of parent –child attachment. In the COS intervention graphical illustrations of “the Circle of Security” are used. This Circle is a “roadmap” that encompasses the three basic control systems; the attachment system, the exploration system and the caregiving system [62]. The parent is illustrated through the pair of hands that hold together the child’s world. In COS concepts, “holding” means to serve as a secure base and safe haven [63]. “The top half” of the Circle depicts the child’s exploration system and needs. I order to explore, the child needs the parent to serve as a secure base by “watching over,” “delighting in,” “helping,” and “enjoying with” the child. Having a parent that supports exploration helps the child develop his or her own sense of interest, leading to mastery and competency in later years. Along “the bottom half” is the child’s attachment needs: “protect me,” “comfort me,” “delight in me,” and “organize my feelings.” By delighting in the child, the parent helps the child constructing an internal representation of him- of herself as a loved person and thereby establish self-worth, and by organizing the child’s feelings by accepting, sharing and naming them, the parent co-regulates the child’s emotions and lays the groundwork of later self-regulation of emotions [63]. The child’s needs for comfort and exploration encouragement shift rapidly and the caregiver must continuously adjust to those needs, whenever possible.

Evidence from a meta-analysis shows that attachment security can be effectively influenced by interventions that target parental, especially maternal sensitivity, especially when conducted in at-risk samples. Further, it is found that short term group approaches (<16 sessions) are most effective [64]. The program “Circle of Security Parenting” (COS-P) is a recent and shorter version of COS that consists of minimum 8 weekly sessions of 1½ to 2 hours durations without the initial individual video assessment of attachment, which is part of the longer COS program. In COS-P standard video materials of child attachment behaviors as well as the graphic materials to illustrate the Circle of Security are used. The manual is structured in eight chapters each focusing on a specific theme, such as “The Circle of Security”, “Exploration of the child’s needs in the circle”, “Being with your child in the circle”, “Exploration of own challenges in meeting child’s needs”, “Disruption and repair of the relationship”. At least one session is used per theme although in many settings it may be recommended to spent more time on some of the chapters in the manual, thus the duration of the program may be somewhat extended.

To the best of our knowledge, the effectiveness of COS-P used in a community setting as an indicated intervention program for at–risk families has not been fully tested in an larger RCT design, making this study the first of its kind. Results from this study will provide new evidence regarding the efficacy of COS-P, a program developed in the Unites States, when implemented as a short term indicated parenting group program in a Scandinavian country.

### Objectives and specific hypotheses

The aim of this study is to determine whether COS-P as an indicated short group-based educational intervention can lead to.

### Primary outcome

Improved maternal sensitivity

### Secondary outcomes

- More securely attached infants
- Reduced maternal depressive symptoms
- Improved maternal ability to mentalise
- Reduced parental stress and improved family functioning
- Improved infant cognitive, language and socioemotional development
- Heterogeneity of effects across family type with disadvantaged families gaining more from the intervention

## Methods

### Trial design

In a parallel randomized controlled trial of two intervention groups this study tests the efficacy of the program Circle of Security- Parenting (COS-P) compared to Care as Usual (CAU) in enhancing maternal sensitivity and child attachment in a community sample identified to be at-risk in the City of Copenhagen, Denmark. During the project period (2015–2019) a general population of an estimated 17.600 families with an infant aged 2–12 months are screened for the infant mental health risk factors maternal postnatal depression and infant social withdrawal. A total of 314 eligible families, who agree to participate, will randomly be allocated with a ratio of 2:1 into the COS-P intervention arm and into CAU. The goal is that at least 250 families will complete follow-up.

### Study setting

The study is conducted in collaboration with the central administration and the community health nurses in the City of Copenhagen. Danish national guidelines comprise an extensive level of universally available nursing support to families with new born babies [65]. Since 1974, under the Act on the Danish Home Visiting Program, regular examinations in infancy are performed by health nurses in the infant’s home, including measuring growth of the head, length and weight, evaluating motor and speech development, guidance of infants’ emotional and developmental needs. The Danish home visiting program is very well accepted by parents and only 1–2 families out of 1000 reject contact with the health nurse [66].

During the first year of the child’s life the health nurse in the City of Copenhagen visits the family and examines the infant at least twice within the first 3 weeks after birth, at 2 months, at 4 months (only first time mothers) and at 8 months. During the project families identified to be at risk either due to maternal postnatal depression and/or infant social withdrawal will be randomized to either Care as usual (CAU) in the City of Copenhagen or Circle of Security – Parenting (COS-P). Referral to the project is not possible before the 2 months visit.

The COS-P intervention will take place at Babylab, University of Copenhagen which has a very central location near to public transportation. The Babylab has full access to large therapy rooms for conducting the COS-P groups, as well as rooms to be used for babysitting. For the follow-up visit to assess outcome measures, the Babylab is a fully equipped observational lab that offers all facilities needed for the assessments planned for the study. The lab has an observational room with a one-way screen, modern cameras and video-recording equipment to tape observational assessments.

### Participants

Eligible participants are mothers and their partners living in Copenhagen with an infant aged 2–12 months born at term (Gestational age (GA) 37–42) or born preterm (GA 30–36).

### Inclusion criteria for participants

- Mother is ≥ 18 years old and speaks and understands Danish.
- Mother is screened positive for symptoms of postnatal depression (EPDS ≥10) and fulfill criteria for a diagnosis of depression assessed in a clinical interview (SCID-5/RV) conducted by a psychologist trained in the SCID-5 10–20 days after the initial EPDS screening AND/OR
- Infant is scored to be socially withdrawn in two ADBB assessments (ADBB ≥5) conducted within a range of 10–20 days when the infant is 2, 4 or 8 months.
- If there is a father/partner this person speaks and understands Danish or English.

### Exclusion criteria for participants

Infant severe medical condition, known autism and/or early retardation, maternal bipolar disorder and/or psychotic disorder, known severe intellectual impairment, suicide attempt during pregnancy or postnatally and/or present alcohol/substance abuse. Furthermore, families will be excluded, if they express that they intend to move away from the Copenhagen area within the period of the intervention.

The health nurses in the project who conduct the screening for postnatal depression (EPDS) and infant social withdrawal (ADBB) are familiar with the eligibility criteria of CIMHP. In the Danish system families meeting any of the exclusion criteria are most likely to be known already if the mother has participated in any antenatal examination at the GP or at the midwife. These families at severe risk will be enrolled in the treatment as usual in the City of Copenhagen, which includes e.g. psychiatric treatment, treatment for substance abuse, hospitalization etc.

### Interventions

#### Circle of security-parenting (COS-P)

The COS-P manual and video material has been translated to Danish (Tryghedscirklen – Forældreprogrammet, manual, Lier, 2013). Based on standard video material of parent-infant interactions, parents are trained to see and understand infant attachment behavior and especially to learn about infant miscuing attachment signals. In the COS-P intervention graphics and video illustrations is designed in a pedagogical form with the aim of meeting the variability of participants in motivation, requirements, openness and compliance with treatment. In the current study, the intervention consists of 10 weekly 90 min sessions, as more time is spend on chapter three and five in the COS-P manual. Both mother and her partner are invited to participate and each group includes 5–7 families. Child minding facilities are provided during the sessions. The families who are allocated to the COS-P intervention are not excluded from receiving other treatment, for example antidepressant medication, psychotherapy, and/or CAU as well. If a COS-P family experiences a crisis they may be offered extra home visits by the health nurse. The health nurse of the family remains to be the primary responsible person of the family and she will by default continue to pay both the COS-P and CAU families the routine health visits.

### Adherence

The psychologists conducting the COS-P intervention are all certified in COS-P. All COS-P group sessions will be videotaped and coded for therapist integrity and adherence to the COS-P manual using a COS-P session checklist. Moreover, to ensure adherence to the manual, and to the COS-P approach, the treatment team receives regularly supervision (via Skype) from a supervisor appointed by the developers of the COS-P.

### Care as usual (CAU)

The existing standard practices for infants and families at risk in Copenhagen will be the active control condition. These vary in content and duration in the districts of Copenhagen. Likewise, CAU may change during the project period. At project start all districts offered (a) group interventions for mothers who experience postnatal depressive symptoms and/or (b) extra counselling home-visits by a health nurse. Number and content of extra home-visits vary in accordance with the families’ specific needs, but rarely exceed 12 extra visits per year. Furthermore, all districts also have offers to families who experience different kinds of parenting difficulties (not specified) in the postpartum period. For example, The Incredible Years, Parents and Babies®, a group-based 10-session intervention for mothers and infants, and individual MARTE-MEO® intervention. Finally, the nurses can refer the family to anonymous counselling provided by the local social security services (“Anonym rådgivning, Familiehuset, Socialforvaltningen”).

When a family is allocated to CAU, UCPH Babylab informs the nurse who referred the family to the project, and she contacts the family and discusses with the family what type of CAU intervention is appropriate for the family. Every third months, staff from Babylab will contact the health nurses who have families referred to the CAU-group to monitor the CAU group with respect to what specific CAU intervention the families have been offered, compliance, drop-out rates etc.

### Measures

There are two points of assessments for COS-P and CAU groups: At baseline (T0) when the infant is 2–12 months old and at follow-up (T1), when the infant is 12–16 months. T0 takes place at a visit in the families’ homes. T1 takes place at UCPH Babylab for both COS-P and CAU groups. To promote retention and complete follow-up participants will receive a gift card of 200 DKK when completing follow-up assessments.

### Background information and control variables

Information about risk condition (infant social withdrawal, maternal depression, or both), infant gender and infant age at referral, as well as parent age, gender, marital status, educational background, employment status, current and lifetime depression status, parental attachment style, personality dysfunctioning, family functioning, alcohol and drug abuse, smoking, parental adverse childhood experiences will be collected through surveys at baseline. Basic background information on eligible individuals who are not enrolled in the study is available from register-based data. Furthermore, we ask decliners about their reason for decline

### Primary study outcome

*Maternal sensitivity* is the core experimental variable that COS-P aims to enhance. Sensitive responses, the ability to respond appropriately to the child’s attachment needs [67], has consistently been found to be the most reliable predictor of attachment security [64, 68, 69]. Maternal sensitivity is observed during 5 minutes mother-infant interaction (free play), and will be assessed during the home-visit at T0 and during the lab-visit at T1. The “Coding Interactive Behavior” (CIB) [70] will be used to code maternal sensitivity. The CIB is a global rating system for social interactions that includes 52 codes rated on a scale of 1 to 5 which are aggregated into several composites. The system has been validated in multiple longitudinal studies of normative and high-risk populations in infancy, preschool, and adolescence interacting with mother, and has shown adequate psychometric properties, including construct validity, test–retest reliability, and predictive validity [71–75]. All mother-infant interactions will be video recorded and coded by reliable coders blind to treatment allocation and with no clinical involvement in the study. Inter-coder agreement will be calculated on a randomly selected subset of 20% that will be coded by another reliable coder blind to treatment allocation.

### Secondary study outcomes

*Infant*-*mother attachment quality* is the second core experimental variable in COS-P. Infant-mother attachment is generally thought to reflect how well toddlers are functioning in the relationship with their primary caregiver. Moreover, infant-mother attachment quality has been documented to play a crucial role in the child’s subsequent social and emotional development [6, 10, 11, 76]. Infant-mother attachment is observed at T1 in UCPH Babylab and assessed with The Strange Situation Procedure (SSP) [19, 20]. SSP is the most widely used and well-validated experimental paradigm for assessing the quality of the child’s attachment to a parent in infancy [10, 11, 76]. From the SSP, that is being video recorded, the child is observed in eight consecutive brief episodes that are designed to evoke mild stress to trigger the attachment behavior of the child. During the eight episodes (each of a maximum duration of 3 minutes) the mother and child are introduced to an unfamiliar room. Then a stranger enters the room and the child is separated two times from his/her mother as the mother leaves the room. The child’s attachment behavior is coded from the reunion episodes based on four interactive behavior scales: proximity-seeking, contact-maintaining, avoidance of the caregiver and resistance.

Continuous measures of attachment security and disorganization (in both infants and adults) have been suggested to be better suited than the categorical measures when subtle differences in attachment security and disorganization cannot be detected using the categorical approach [77–79]. Following IJzendoorn and Kroonenberg [80], we will therefore calculate a continuous attachment score from the four interactive scales used for the classification of the conventional attachment categories. Higher scores indicate more attachment security. This approach has been further validated in recently published studies [81, 82]. Continuous scores for disorganization will be derived directly from coding the conventional 9-point Disorganization scale [20] with higher scores indicating more disorganized behavior.

SSP will be conducted by trained experimenters, and will be video recorded from three angles to facilitate coding. Attachment behavior will be coded from video-recordings by a coder trained at the University of Minnesota, who is blind to group status and has no clinical involvement in the study. For inter-coder agreement, a randomly selected subset of 20% of the SSPs will be coded by a second coder, also trained at University of Minnesota, blind to group status and with no clinical involvement in the study.

*Infant social withdrawal* at T0 and T1 will be assessed with the Alarm Distress Baby Scale, ADBB [83]. The ADBB is an observational instrument with eight items related to the infant’s social behavior. It is used during a routine physical examination of the infant aged 2–24 months where the clinician, e.g. the healthcare nurse, engages with the infant. The eight items, each rated from zero to four are: facial expression; eye contact; general level of activity; self-stimulation gestures; vocalizations; briskness of response to stimulation; relationship to the observer, and attractiveness to the observer. The clinician keeps in mind the eight items while conducting the routine physical assessment, and then spends approximately 5 minutes completing the scale. Low scores being optimal social behavior, and a cut-off ≥5 is recommended. In infant cases scoring ≥5, the ABDD observation is recommended to be conducted again after 2 weeks to assess whether the social withdrawal is persistent [24]. In a recent review [22] of 13 studies using ADBB, the scale has been found to show good psychometric properties as well as good inter-rater reliability (>.70) and acceptable test-re-test reliability. The test-retest stability is found to be .84 -.90 and its internal consistency is found to be satisfactory (Cronbachs alpha = .83). Using the ADBB in more countries, a prevalence of around 4% of socially withdrawn infants has been found [25, 27]. According to the ADBB-manual, the ADBB can be coded from a variety of situations, and in the present study, infant social withdrawal will be assessed during the BSID-III assessment (see below). ADBB assessment will be conducted by a psychologist who is certified as a reliable ADBB-coder by Dr. Guedeney. All assessments will be video recorded, and for inter-coder agreement, a subset of 50% of the assessments will be randomly selected and coded by a second coder blind to group status.

*Levels of maternal and partner mentalization* will be assessed (T0 and T1) using The Parental Reflective Functioning Questionnaire-1, PRFQ-1 [84]. It consists of 39 items comprising three sub-scales prototypically describing high, low, and neither high nor low mentalizing in parents. Scoring procedures precepts yield a total score on all three subscales that assesses parental reflective functioning or mentalizing, that is, the capacity to treat the infant as a psychological agent. Preliminary validation studies of PRFQ-1 have 1) investigated the factor structure, reliability, and relationships of the PRFQ with demographic features, symptomatic distress, attachment dimensions, and emotional availability; 2) the factorial invariance of the PRFQ in mothers and fathers and relationships with demographic features, symptomatic distress, attachment dimensions, and parenting stress were investigated and 3) the relationship between the PRFQ and infant attachment classification as assessed with the Strange Situation Procedure (SSP). Overall, results provide initial evidence for the reliability and validity of the PRFQ. [85]. Exploratory and confirmatory factor analyses suggested three theoretically consistent and clinically meaningful factors, which were invariant across the two samples and across mothers and fathers, assessing (a) pre-mentalizing modes, (b) certainty about the mental states of the infant, and (c) interest and curiosity in the mental states of the infant. These subscales had good internal consistency, were not or only modestly related to demographic features, and were generally related in theoretically expected ways to parental attachment dimensions, emotional availability, parenting stress, and infant attachment status in the SSP.

For the present study, the PRFQ has been translated with permission from the authors according to scientific standards, with two independent translations which were compiled, pilot tested in a sample of 12 Danish parents, adjusted after interviewing these parents, back-translated by a native English speaker blind to the original version, and back-translation finally approved by Patrick Luyten (January 2015).

*The Parenting Stress Index*, *Third Edition*, *PSI* [86], Danish version, Hogrefe Forlag will be used to assess distress in relation to caregiving and the relation to the child (T0 and T1). The PSI is designed for the early identification of parenting and family characteristics that fail to promote normal development and functioning in children, children with behavioral and emotional problems, and parents who are at risk for dysfunctional parenting. It can be used with parents of children as young as 1 month. The PSI manual states that PSI was developed on the theory that the total stress a parent experiences is a function of certain salient child characteristics, parent characteristics, and situations that are directly related to the role of being a parent. The PSI identifies dysfunctional parenting and predicts the potential for parental behavior problems and child adjustment difficulties within the family system. The PSI consists of 120 items and can be completed by parents in less than 30 min. The results of the completed PSI are a Total Stress Score, plus scale scores for both Child and Parent Characteristics. The child characteristics are measured in six subscales: Distractibility/Hyperactivity, Adaptability, Reinforces Parent, Demandingness, Mood, and Acceptability. The parent personality and situational variables component consists of seven subscales: Competence, Isolation, Attachment, Health, Role Restriction, Depression, and Spouse. The PSI has been empirically validated to predict observed parenting behavior and children’s current and future behavioral and emotional adjustment in many cultures [86].

*The Ages and Stages Questionnaires* –*Social*-*Emotional*, ASQ-SE [87] will be used to asses infant socio-emotional development at T1. Both mothers and fathers will be asked to fill in the questionnaire, and maternal and paternal report will be analyzed separately. Domains being assessed are Self-regulation, compliance, communication, adaptive functioning, autonomy, affects and interaction with people. The ASQ-SE is developed as a screening instrument, but is also used for monitoring progression. For the present study, the ASQ-Se has been translated with permission from the authors according to scientific standards, with two independent translations which were compiled, pilot tested in a sample of Danish parents, adjusted after interviewing these parents, back-translated by a native English speaker blind to the original version, and back-translation finally approved by Brooks Publishing.

*Bayley Scales of Infant and Toddler Development 3rd Edition* -*Screening Test*, *BSID III* (Pearson, 2008) will be used to assess infant cognitive development (T0 and T1) and infant language development (T1). The BSID is a standardized norm-based test widely used to assess general indices of infant mental development. The cognitive scale assesses memory and problem solving, exploration and manipulation, object relatedness, and sensorimotor development. The language scale is a composite of two subscales: an expressive scale (babbling, gesturing and utterances) and a receptive communication scale (verbal comprehension and vocabulary). Raw scores for each subscale are converted into scaled scores (range 1–19, *M* = 10, *SD* = 3), and a composite score (*M* = 100, *SD* = 15) can be derived from the scaled score for cognitive development, and the sum of the two language scaled scores. The test will be administered by trained psychologist who are routinely supervised based on video recordings of the tests. For inter-rater agreement, a randomly selected subset of 50% of the tests will be coded from video-recordings by a trained psychologist blind to group status and with no clinical involvement in the study.

*Edinburg Postnatal Depression Scale*, *EPDS* [45] Cox et al, 1987) will be used to assess maternal depressive symptoms at T0 and T1. The effectiveness of EPDS for detection of women at risk for or suffering from PND at a clinical level is well-documented, and across countries the EPDS has been shown to have a high sensitivity (68–95%) and high specificity (78–96%) against a clinical psychiatric diagnosis of depression [46–50]. EPDS includes comprises questions with four possible responses related to mood and feelings. Total score ranges from 0 to 30 points. Scores in the range of 0–9 are considered as indicating the presence of symptoms of distress that may be short-lived. Scores from 10 to 12 are considered to indicate probable depression, and further assessment is recommended. Scores equal to or above 13 are considered to indicate the presence of depression [45, 50].

*Structured Clinical Interview for DSM*-*5 disorders* - *Research Version*, *SCID*-*5*-*RV* [88] is a semi-structured interview guide for systematically making DSM-5 diagnoses. It will be administered by a trained research psychologist who is routinely supervised based on sound recordings of the interview. At T0 SCID-5-TR will be used to assess maternal current and past major depressive episode (MDE), current psychological and psychiatric treatment status, current and past alcohol and substance abuse, current and past bipolar disorder, current and past suicidal symptoms, as well as psychotic symptoms, using the following modules: (1) Overview, non-patient Version. (2) Module A. 4.b. Mood Episodes; (3) Module B, 5b Psychotic Screening; (4) Module E, 7. Alcohol and substance Use Disorders. At T1 SCID-5-RV, Module A, will be used to assess current MDE. For inter-rater agreement, a randomly selected subset of 50% of the interviews will be coded from sound recordings by a trained psychologist blind to group status and EPDS-score.

*Hopkins Symptom check list*, *SCL*-*92* [89] will be used to assess maternal and partners overall level of symptom severity at T0 and T1. SCL-92 is a multidimensional self-report symptom inventory for measuring current psychological distress or the degree of affective distress. The SCL-92 version used in this study is a combination of the SCL-90 and SCL-90-R, and the validity of SCL-92 has been demonstrated in a Danish population by Mokken-Loevinger analysis and Rasch analysis [89]. SCL-92 covers nine different dimensions of mental distress: somatization, interpersonal sensitivity, depression, anxiety, phobic anxiety, obsession-compulsion, hostility, paranoid ideation, and psychoticism. Scoring results in both a symptom profile and a general distress score (Global severity Index, GSI). The questionnaire comprises 92 items which are rated on a five-point Likert Scale ranging from 0 (not at all) to 4 (extremely). The timeframe is the past week.

*Experience in Close relationships* – *revised version*, *ECR*-*R* [90] will be used to assess participants attachment (T0 and T1). This is a 36 items questionnaire measuring adults attachment in close relationships. It is the most frequently used self-report measure of adult attachment in the international literature. The ECR has good psychometric properties [91]. It measures (a) attachment avoidance, which is characterized by a fear of intimacy and interpersonal dependence and (b) attachment anxiety, which is characterized by fear of abandonment and a craving for interpersonal closeness. Avoidance and anxiety are continuous dimensions with attachment security defined as the absence of both.

*The McMaster Family Functioning Device*, *FAD* [92], Danish version [93] will be used to assess Family functioning as reported by mother and partner (T0 and T1). In the present study the General Functioning subscale of the Family Assessment Device (FAD-GF) will be used. FAD-GF assesses overall healthy functioning or dysfunction of intrafamilial relationships. The scale was derived by summing items that sampled the 6 domains included in the McMaster Model of Family Functioning: problem solving, communication, roles, affective responsiveness, affective involvement, and behavioral control. Higher scores indicate greater family dysfunction. FAD has been reported to have good psychometric properties, and to be a reliable and valid assessment of both clinical and non-clinical families [94].

*State*-*Trait Anxiety Questionnaire* (*STAI*) [95] will be used to assess maternal and partner’s level of anxiety at T0 and T1. STAI is a commonly used measure of trait and state anxiety. It can be used to diagnose anxiety and to distinguish it from depressive syndromes, and in the present study, it will be used to distinguish mothers suffering from PND with and without co-morbid anxiety. It is also often used as an indicator of caregiver distress [96, 97] which also will be the case in the present study. The questionnaire has 20 items for assessing trait anxiety and 20 for state anxiety. All items are rated on a 4-point scale. Higher scores indicate greater anxiety. Internal consistency coefficients for the scale have ranged from .86 to .95; test-retest reliability coefficients have ranged from .65 to .75 over a 2-month interval (Spielberger et al., 1983). Considerable evidence has demonstrated the construct and concurrent validity of the scale [98].

*Standardized Assessment of Personality* – *Abbreviated Scale*, *SAPAS* [99] will be administered at T0to assess level of personality dysfunctioning in both mother and partner. This is an eight item screening interview for personality disorder/personality dysfunctioning. Each item is worded as a question to be answered with yes or no (e.g., item 1: “In general, do you have difficulty making and keeping friends?”). When the response is given that indicates pathology (i.e., yes to item 1), the interviewer must follow up by asking if that is true in general. A total score of 3 on the screening interview is considered to indicate the presence of a DSM-IV/5 personality disorder. As the SAPAS is a set of indicators covering multiple areas, it is not designed to be unidimensional. Rather, the SAPAS is designed to cover different areas of personality. The sensitivity and specificity of the scale has been found to be 0.94 and 0.85 respectively when validated against a clinical diagnosis of personality assessed in a standardized diagnostic interview [99]. Further evidence of the concurrent and construct validity of the scale has been demonstrated in several studies [100–102].

*Family and Social Support Scale*, *FSS* [103] will be administered at T0 and T1 to assess the extent to which the mother and her partner experience support from the family, friends, the society and partner. The scale consists of 19 items rated on a 5-point scale ranging from not at all helpful (1) to extremely helpful (5). Scoring results in a total score with higher scores indicating higher levels of support. FSS has been reported to have good psychometric properties; Coefficient alpha for the scale was found to be .79, with split-half reliability of .77 corrected for length [104]. The scale has been validated in a range of cultures, and used in many different studies examining the effect of social support on parent health and wellbeing, family integrity, parental perceptions of child functioning, and styles of parent–child interaction [105–107]. For the present study, the FSS has been translated with permission from the authors according to scientific standards, with two independent translations which were compiled, pilot tested in a sample of 13 Danish parents, adjusted after interviewing these parents, back-translated by a native English speaker blind to the original version, and back-translation finally approved by Carl Dunst (April 2015) Table 1.

**Table 1:** Points of measurements of primary and secondary outcomes

Measure	Baseline (Infant age 2–12 months)	Follow-up (Infant age 12–16 months)
Maternal Sensitivity (CIB)	X	X
Infant-Mother Attachment Quality (SSP)		X
Maternal Parenting Stress (PSI)	X	X
Maternal reflective functioning (PRFQ)	X	X
Infant Social Withdrawal (ADBB)	X	X
Infant socio-emotional development (ASQ-SE)		X
Infant cognitive development (BSID-III)	X	X
Infant language development (BSID-III)		X
Family Functioning (FAD)	X	X
Maternal experience of support (FSS)	X	X
Maternal Depressive symptoms (EPDS)	X	X
Maternal Depression status (SCID-5-RV)	X	X
Maternal overall psych. distress (SCL-92)	X	X
Maternal attachment (ECR)	X	X
Maternal Anxiety (STAI)	X	X

### Sample size

Based on a literature review regarding assessment of maternal sensitivity using CIB (Feldman, 1998) conducted by Dr. Væver it was assumed for the power analyses that the average maternal sensitivity score at baseline is 3 with a standard deviation of 0.9. Mothers will be tested both at baseline (T0) and at follow-up (T1). The primary comparison of COS-P and CAU will be adjusted for baseline scores. In the power analysis we will assume a test-retest correlation of 0.5. Ignoring clustering due to treatment groups in the data a sample of 200 dyads would provide 90% power to detect a treatment effect of around 0.40. The final statistical analysis will take clustering into account, but as there are not prior studies to pin down the intra-group effect a conservative approach will be employed. To accommodate this we will aim for having 250 dyads in the final analysis, which is deemed more than sufficient to handle any plausible clustering effect. A likely drop-out for 20% (either during the intervention period or at follow-up) brings the final sample size to 314 at time of randomization. Due to the nature of the trial there are no planned interim analyses or early stopping rules.

### Recruitment plan and expected participant timeline

The project is estimated to run over a 5 years period (2015–2019). During the project period an estimated 17.600 mother-infant dyads will be screened by community health nurses using two standardized screening instruments:

1. The Alarm Distress Baby Scale (ADBB) in detecting infant social withdrawal at 2, 4 (only first time mothers) and 8 months With a cut-off score of 4/5 and an expected prevalence of 4% [22] we expect the nurses to identify 704 infants scoring above cutoff during the project period. We expect that up to half (*n* = 352) of these families will refuse to be referred to the project, and/or will fulfill exclusion criteria (e.g. mother does not understand and speak Danish, the mother has psychotic symptoms etc.).
2. The Edinburgh Postnatal Depression Scale (EPDS) in detecting maternal postnatal depression 2 months postpartum. With a cut-off score of 10/11 and a point prevalence of 5.5% [108] we expect the nurses to identify 968 mothers with depressive symptoms. Again, we expect that up to half (*n* = 484) of the families will refuse to be referred to the project and/or fulfill exclusion criteria.

Confirmation of infant social withdrawal and depression status will be conducted by clinical psychologists during a home visit (T0) 10–20 days after the health nurses’ screening.

**Fig. 1:**
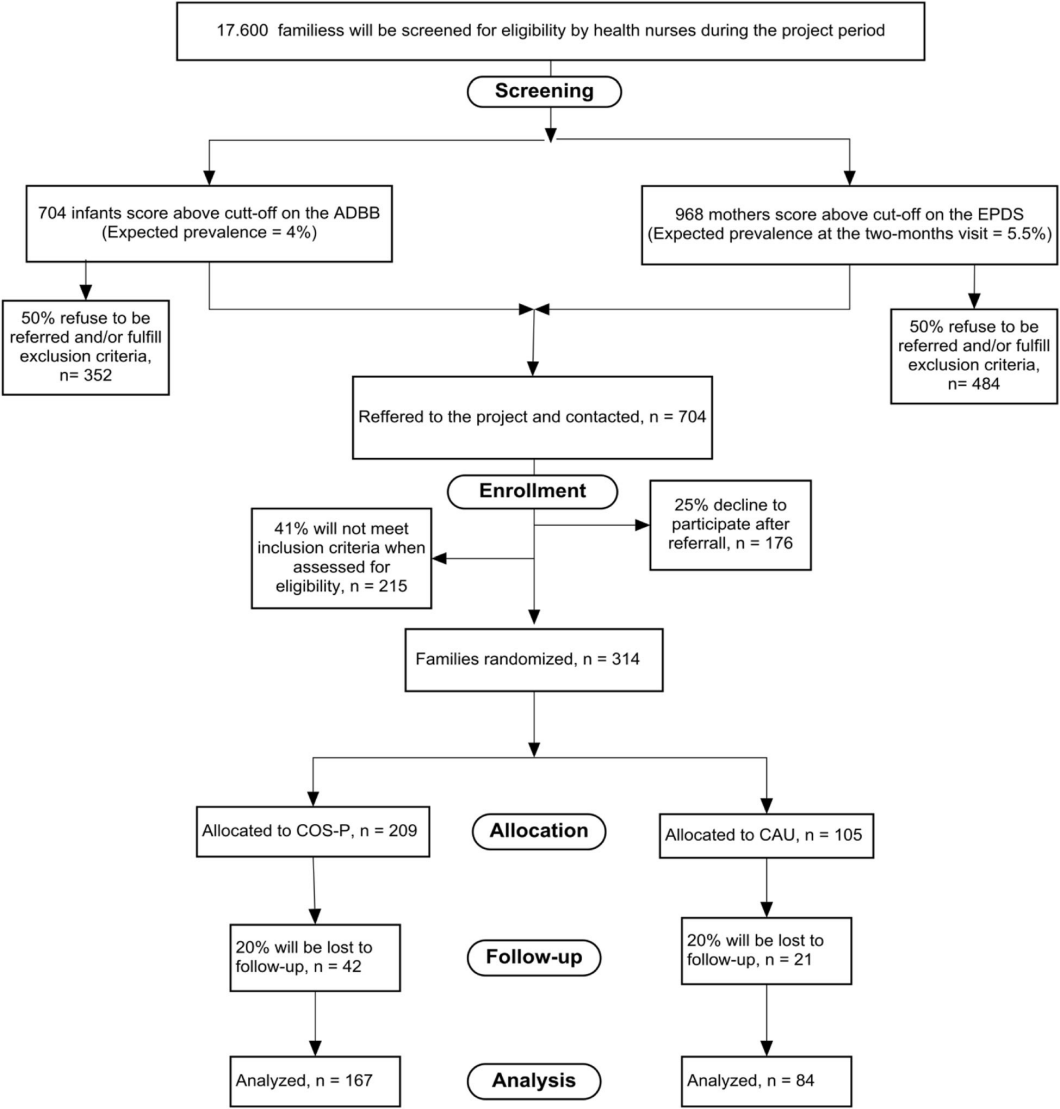
Enrollment chart for CIMHP

In total, we expect that 704 families agree to be referred. Of these families, we expect that up to 25% will decline to participate either after being contacted or during baseline assessments. Moreover, for up to 41% of the families, we expect that inclusion criteria will not be fulfilled/exclusion criteria will be fulfilled at T0 (e.g. the mother does not meet criteria for major depressive episode, infant social withdrawal is not confirmed, mother fulfill criteria for bipolar disorder etc.) See Fig. 1.

A sample of an estimated 113 eligible parent (s) from the ADBB screening and an estimated 201 eligible parent (s) from the EPDS screening will enter into the clinical, randomized controlled trial to test the efficacy of the group counseling program (COS-P) compared to Care as Usual (CAU). Intake to the RCT will stop when the sample of 314 has been enrolled (see enrollment chart, next page). We aim for 250 families to complete the follow-up (see sample size).

### Randomization

Allocation ration is 2:1 to either COS-P or CAU. For allocation of participants, a computer-generated list of random numbers is used. The list is created using block randomization with random block sizes of 2, 4, or 6. The allocation sequence is generated by an investigator with no clinical involvement in the trial, Associate Professor Theis Lange (TL) and stored in a password-protected electronic document accessible only by TL.

To enter a family into the study, the psychologist who conducts baseline assessments and enroll the families into the trial will open an opaque and sealed envelope containing a card with details of with intervention group the family is randomized to as well as an randomization number. This envelope is prepacked by an assistant with no affiliation to or knowledge about the project who receives the allocation sequence from TL and numbers 450 envelopes sequentially. The packed envelopes are stored with the project coordinator of UCPH BabyLab, to whom the allocation sequence is thus concealed.

When a family is referred to the study by a health nurse, the project coordinator who schedules a home-visit will provide the psychologist with the next consecutively numbered envelope to bring to the visit. To prevent subversion of the allocation sequence, the lab-coordinator will write the name and date of birth of the child on the envelope when a visit is scheduled, and a video-recording is made of the sealed envelope with participant details visible. A second researcher will later view the recordings to ensure envelopes are still sealed when participants’ names are written on them. If a family is not included during the home-visit, the envelope is given back to the lab-coordinator who will store all none-opened envelopes, and the randomization number is not used. Unused envelopes are not reused purely for logistic reasons.

### Statistical methods

The analysis of both primary and secondary endpoints will be conducted under the intention to treat principle. However, as a supplement we will also conduct analyses where the actual treatment participation is taken into account. Intuitively the first analysis provides as lower bound for the effect of the COS-P treatment (as it evaluates a likely unfavorable condition) while the second analysis provide an upper bound (as it evaluates a likely favorable condition). If more than 95% observations are complete we will only conduct a complete case analysis. Otherwise multiple imputation will be used to handle missing data. Imputations will be conducted using the software package REALCOM [109], which includes an extension of the chained-equation technique to clustered data.

The primary comparison of COS-P and CAU is conducted using linear regression of maternal sensitivity at T1 on treatment group adjusted for baseline maternal sensitivity. Generalized estimating equations (GEE) will be employed to account for the correlation induced by mothers attending the same group in the COS-P arm, the same therapist treating several mothers, and similar in the CAU group. A two sided test with a 5% significance level will be employed. When estimating the effect size the model will further be adjusted for a) the T0 values of maternal sensitivity and b) the family characteristics mentioned above; Both will be reported but the value a will be considered the important. To assess the degree to which the treatment effect is diluted by the inclusion of non-completers (mothers participating in 4 or fewer sessions) we will repeat the analyses above, but excluding non-completers from the COS-P group.

Secondary endpoints where a baseline value is available will be analyzed in the same way as the primary endpoint, see above. Binary outcomes will be analyzed by logistic regressions and continuous outcomes by linear regressions. For secondary endpoints where no baseline is available the analyses will only be adjusted for the family characteristics mentioned above. GEE techniques will be used throughout Significance will be assessed both using a 5% significance level and adjusted for multiple testing (6 tests planed) by the Holm–Bonferroni method. Findings significant even after correcting for multiple testing will be treated as truly significant findings while findings with *p*-values below 5%, but not significant after correcting for multiple testing will be described as potential significant findings.

The final secondary endpoint on heterogeneity of effects of COS-P across family types will be assessed by including an interaction term between group membership and family type (family types are: 1) Infant socially withdrawn; 2) Infant socially withdrawn AND maternal depression; and 3) Maternal depression only in the linear regression planned for the primary analysis.

For both logistic regressions and linear regressions model fit will be assessed by graphical procedures. In case of poor model fit additional sensitivity analyses and bootstrap procedures will be employed to establish if the findings of the study are sensitive to the lack of model fit. A detailed statistical analysis plan will be completed before half of the intended sample size has been recruited.

## Discussion

The protocol describes an experimental evaluation of an indicated brief manual- and group-based parenting educational program to enhance parental sensitivity and attachment compared to care as usual in a large community sample. This is an evaluation that has not yet been made in Denmark or internationally. Results will provide new evidence regarding the efficacy of a short term indicated parenting group program developed in the Unites States when implemented in a Scandinavian country. Further, COS-P is a promising approach as health nurses can be trained COS-P therapists in a future up-scaling. The efficacy of COS-P will be compared to the efficacy of Care as Usual (CAU) offered in Copenhagen to families identified to be at risk and in need of support. Results from this study will inform the City of Copenhagen of whether offering a systematic manual based short term parent intervention is more efficient in targeting infant mental health risks than what is currently offered as indicated prevention. Further, as we are collaborating with economist the study will provide knowledge of the cost-effectiveness of COS-P compared to CAU in the City of Copenhagen. If proved effective the study will represent a notable advance to initiating the COS-P intervention as part of a better infant mental health strategy in Denmark. Conversely, if this system is similar or inferior to the current system, this is also important knowledge in regard to preventing infant mental health risks in a cost effective way in a general population.

### Harms

The program consists of ten group 90 min COS-P sessions at UCPH Babylab. All participants are parents being challenged by maternal depression and/or infant social withdrawal in the first year of their baby’s life. During the period of COS-P intervention the mothers are assessed with EPDS to monitor her level of depression. Likewise, the infants showing signs of infant withdrawal are assessed with the ADBB to monitor the level of social withdrawal during the intervention period. At least for some individuals, the participation in a group may be experienced as a challenge, as well as the level of time consume to participate in COS-P may be a challenge for some. Most sessions are held late afternoon to promote the participation of the partners, as well as child minding facilities are offered during the COS-P sessions to ease the practical challenges of the families’ participation in COS-P. Further, as previously explained, participation is entirely voluntary and a decline to participate does not in any way affect assess to family services provided by the municipality. For these reasons, we expect the intervention to be associated with very low risk for participants.

### Registration

The project is registered with ClinicalTrials.gov: ID: NCT02497677. Registered July 15 2015

## Abbreviation

CIMHP: Copenhagen infant mental health project
